# Pulsed radiofrequency treatment of the superior hypogastric plexus in an interstitial cystitis patient with chronic pain and symptoms refractory to oral and intravesical medications and bladder hydrodistension: A case report

**DOI:** 10.1097/MD.0000000000005549

**Published:** 2016-12-09

**Authors:** Jong Hae Kim, Eugene Kim, Bong Il Kim

**Affiliations:** Department of Anesthesiology and Pain Medicine, School of Medicine, Catholic University of Daegu, Daegu, Republic of Korea.

**Keywords:** interstitial cystitis, pulsed radiofrequency therapy, superior hypogastric plexus

## Abstract

**Rationale:**

A variety of therapeutic modalities are available for the treatment of interstitial cystitis. However, among them, the less invasive therapies are usually ineffective, whereas the invasive ones carry potential risks of serious side effects and complications. Pulsed radiofrequency (PRF) treatment of the superior hypogastric plexus may be an alternative to conventional treatments, as it provides nondestructive neuromodulation to the superior hypogastric plexus, which transmits the majority of pain signals from the pelvic viscera.

**Patient concerns:**

For 7 years, a 35-year-old female patient had been experiencing lower abdominal pain provoked by urinary bladder filling, perivulvar pain developing spontaneously during sleep or upon postural change, urinary urgency and frequency with 15- to 60-min intervals between urinations, and nocturia with 10 voids per night. Hydrodistension of the bladder, monthly intravesical administration of sterile sodium chondroitin sulfate, and oral medications including gabapentin and pentosan polysulfate had not been effective in managing the pain and symptoms.

**Diagnoses and interventions:**

Given the satisfactory result of a diagnostic block of the superior hypogastric plexus, 2 sessions of PRF treatment of the superior hypogastric plexus, which applied radiofrequency pulses with a pulse frequency of 2 Hz and a pulse width of 20 ms for 120 s twice per session to maintain the tissue temperature near the electrode at 42°C, were performed at a 6-month interval.

**Outcomes:**

This treatment relieved the pain and symptoms for 2 years and 6 months.

**Lessons:**

PRF treatment of the superior hypogastric plexus results in long-term improvements in the pain and symptoms associated with interstitial cystitis.

## 1 Introduction

Interstitial cystitis is a chronic and debilitating disease characterized by pelvic, perineal, or bladder pain in addition to symptoms of urinary urgency and frequency and nocturia, notwithstanding sterile and cytologically unremarkable urine.^[1,2]^ The diagnosis is usually made by exclusion of other lower urinary tract diseases,^[3]^ and the underlying pathophysiology remains elusive despite extensive research by many investigators. Although multiple therapeutic approaches, such as dietary, behavioral, pharmacological, and interventional therapies, have been attempted, there is still no optimal treatment protocol for interstitial cystitis at present. Recently, however, an American Urological Association guideline that includes 1st- to 6th-line treatment groups based on the potential benefits to patients, the potential severity of adverse events, and the reversibility of the treatment was suggested to provide a clinical framework for the treatment of interstitial cystitis/bladder pain syndrome.^[4]^

The effectiveness of the 1st-line treatments, which are easy to perform, is usually doubtful and supported by limited literature.^[4]^ The oral drug therapies constituting the 2nd-line treatments are more acceptable to patients but have potential side effects that might limit their use and achieve only a modest response.^[5]^ Although intravesical therapies, hydrodistension, neurostimulation, and diversion with or without cystectomy have been recommended for refractory cases, they carry potential risk of an opportunistic infection or might increase treatment costs due to repeated catheterization or anesthesia. Therefore, another therapeutic option that is less invasive and does not cause serious side effects and complications is necessary as an alternative to the above-mentioned treatment modalities. The application of pulsed radiofrequency (PRF), providing nondestructive neuromodulation (avoiding the side effects and complications pertinent to denervation) to the superior hypogastric plexus, which mediates pelvic visceral pain, may be a possible candidate as an alternative treatment. Herein, we report long-term improvements in interstitial cystitis-related pain and symptoms following 2 sessions of PRF treatment of the superior hypogastric plexus in a patient with interstitial cystitis refractory to oral and intravesical medications.

## 2 Case presentation

A 35-year-old female patient (height: 144.6 cm, weight: 53.3 kg) was referred from the urology department to our outpatient pain clinic due to 7-year-long lower abdominal pain provoked by urinary bladder filling. This symptom was relieved by subsequent urination, which was in turn coupled with dysuria. The patient had been diagnosed with interstitial cystitis of an unknown cause at another tertiary hospital 7 years prior and then had been followed up at the urology department for 5 years. She was experiencing the above-mentioned lower abdominal pain, pain in the vulvar region (which developed spontaneously during sleep or upon postural change from sitting to standing), urinary urgency and frequency with 15- to 60-min intervals between urinations, and nocturia with 10 voids per night. Hydrodistension performed 3 months before her visit to the urology department and monthly intravesical administration of sterile sodium chondroitin sulfate solution and oral medications, including gabapentin (150 mg/d) and pentosan polysulfate (300 mg/d) since her visit to the department had not improved her symptoms; in fact, her visual analog scale (VAS) pain score (0 represents no pain, and 10 represents the most severe pain imaginable) remained above 6.

**Figure 1:**
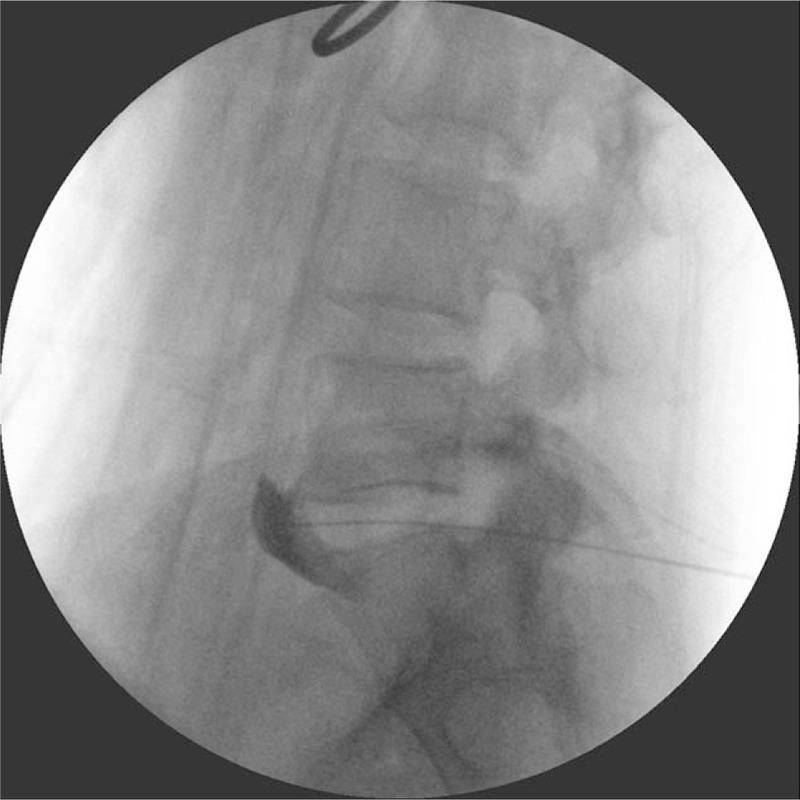
Lateral fluoroscopic view of the lumbosacral spine showing the spread of the radiocontrast anterior to the L5–S1 intervertebral disc.

Therefore, we decided to perform a diagnostic superior hypogastric plexus block under fluoroscopic guidance. To prevent discitis development due to the transdiscal approach, 1 g of ceftriaxone was given intravenously 30 min before the block. The patient was placed in the prone position with a pillow under the lower abdomen to maximize the L5–L1 intervertebral space by flattening the lumbar lordosis. By cephalad tilting the fluoroscopic tube, the double contour lines of the lower endplate of the L5 vertebra were flattened. Then, oblique angulation of the fluoroscopic tube to 15° to 25° to the right side created the optimal trajectory for insertion of a 22-gauge, 150 mm Chiba needle into the L5–S1 intervertebral disc. Fortunately, the iliac crest did not impede the trajectory of the needle. Following local anesthetic infiltration of the skin and subcutaneous tissue 5 to 7 cm from the midline, the needle was introduced via a tunnel vision technique to contact the lateral edge of the right superior articular process of the S1 vertebra. The needle then walked off the superior articular process and was advanced into the L5–S1 intervertebral disc. After a 3-mL capacity syringe filled with 1.5 mL of normal saline was attached to the needle hub, firm pressure was applied to the plunger of the syringe until a loss of resistance was encountered. The spread of the radiocontrast medium, iopromide (Ultravist, Schering AG, Berlin, Germany), anterior to the L5–S1 intervertebral space on the lateral fluoroscopic view of the lumbar spine and within the lateral bony edge on the anteroposterior view confirmed the correct placement of the needle tip near the superior hypogastric plexus (Fig. 1). Subsequent injection of a mixture containing 10 mL of 1% mepivacaine (100 mg) and 1 mL of dexamethasone (5 mg) through the needle relieved the lower abdominal and perivulvar pain (VAS pain score of 2) and reduced the frequency of urination during daytime hours to once every 2 h for 5 h.

**Figure 2:**
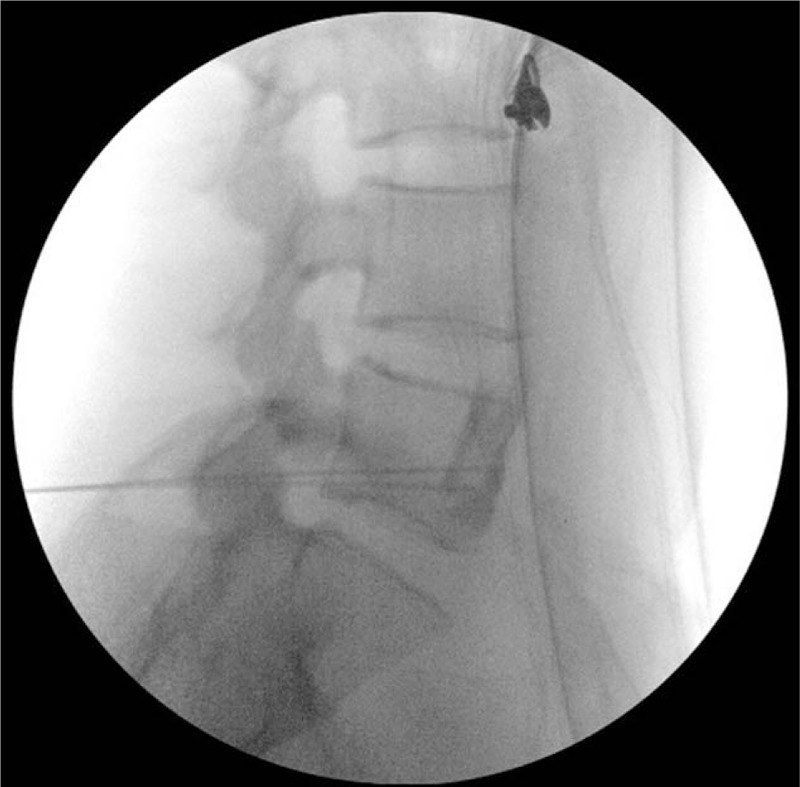
Lateral fluoroscopic view of the lumbosacral spine showing the placement of the bilateral radiofrequency cannulae tips anterior to the L5 vertebral body.

Given the patient's satisfaction with the diagnostic block, PRF treatment of the superior hypogastric plexus was scheduled to be performed 8 days later. After the lumbar lordosis was flattened in the prone position, a disposable 20-gauge, 150 mm, curved radiofrequency cannula with a 10-mm active tip (Model C1510, NeuroTherm, Inc, Wilmington, MA) was introduced 5 to 7 cm laterally from the midline at the level of the L5–S1 interspace and was advanced 45° medially toward the L5 vertebral body under fluoroscopic guidance. The needle tip was then walked off until it reached the anterior aspect of the lower third of the L5 vertebral body on the lateral fluoroscopic view (Fig. 2). A contralateral radiofrequency cannula was inserted using the same technique. The correct placement of the cannulae tips was verified using radiocontrast medium in the same manner as for the diagnostic block. The stylet was then removed and replaced with a radiofrequency electrode. In the absence of a motor response to 2 Hz stimulation at 2 V, radiofrequency pulses (pulse frequency of 2 Hz and pulse width of 20 ms) were applied through the bilateral radiofrequency electrodes, which were connected to a radiofrequency generator (Model PMG-230-TD, Baylis Medical Company, Inc, Montreal, Canada), for 120 s to maintain the tissue temperature near the electrode at 42°C. This procedure was repeated twice on each side. The PRF treatment reduced the VAS pain score to 2 to 3, the urinary frequency interval to 1 to 2 h, and the number of nocturia episodes to 1 void per night; these improvements were maintained for 3 months. However, the VAS pain score was increased to 6 to 8, and the lower urinary tract symptoms (daytime frequency and nocturia) were aggravated again 3 months after the PRF treatment. Although the dose of gabapentin (150 mg/d), which had been maintained during the follow-up at our department, was increased to 300 mg/d, the symptoms did not improve. Another PRF treatment using the same protocol as the 1st PRF treatment was performed 6 months after the 1st PRF treatment, and improvements in the VAS pain score (2 to 3), daytime frequency and nocturia (1 to 2 voids per hour and 1 void per night, respectively) were again achieved. One week after the 2nd PRF treatment, the dose of gabapentin was decreased to 150 mg/d. Until the last follow-up (2 years and 6 months after the 2nd PRF treatment), the reduced dose of gabapentin was continued, maintaining the VAS pain score below 3 as well as decreasing the daytime frequency and nocturia. Because the patient gave written informed consent for the publication of this case report and cannot be identified based on the clinical data in this case report, the approval of the institutional review board was not required.

## 3 Discussion

This case report showed that long-term pain relief and regression of lower urinary tract symptoms were achieved for up to 2 years and 6 months by applying PRF treatment in a patient who had been experiencing lower abdominal and vulvar pain unresponsive to regular intravesical and oral medications.

Although there are many types of treatment for interstitial cystitis, including dietary, behavioral, pharmacological, and interventional therapies, no optimal treatment is presently available. Recently, however, the American Urological Association introduced a guideline for the diagnosis and treatment of interstitial cystitis, which classified treatments into 6 groups according to the extent of invasiveness of the treatments.^[4]^ In the case presented here, the patient experienced failure of treatment with oral medications and hydrodistension, which are the 2nd- and 3rd-line treatments, respectively. Subsequently, we performed a superior hypogastric plexus block and PRF treatment based on the anatomical and physiological aspects of the disease entity, which the guideline does not address, rather than moving to the next treatment step.

As the extension of the aortic plexus, the superior hypogastric plexus is located anterior to the L5 and S1 vertebral bodies and L5–S1 intervertebral disc in the retroperitoneal space below the aortic bifurcation. Caudally, it converges to form the hypogastric nerve that follows the internal iliac vessels and finally becomes the inferior hypogastric plexus. The superior hypogastric plexus and hypogastric nerve predominantly contain sympathetic fibers, which play a major role as a pain pathway from the pelvic viscera, including the urinary bladder. In this context, the superior hypogastric plexus is considered an ideal target for nerve blocking to reduce pelvic visceral pain,^[6]^ and it has been blocked to relieve both benign and malignant pain affecting the pelvic visceral structures.^[6–10]^

However, due to the lack of data on superior hypogastric plexus block for interstitial cystitis, its indication for interstitial cystitis has not been established.^[11]^ Therefore, following a successful diagnostic block, we decided to perform PRF treatment, which is less invasive compared to neurodestructive modalities, which have potential complications and side effects, such as conventional radiofrequency thermocoagulation and neurolysis with alcohol or phenol. In particular, protein coagulation caused by phenol leads to nonselective tissue destruction and the initiation of Wallerian degeneration in nerve fibers, with a neurolytic effect that lasts for several months. However, phenol's toxic effects on the vasculature prevented us from using it to denervate the superior hypogastric plexus because of the anatomical proximity of the superior hypogastric plexus to the distal aorta and common iliac arteries and the theoretically higher risk of neuroma formation resulting from the destruction of the basal neurolemma.^[12]^ Similarly, alcohol induces Wallerian degeneration, with an effect that lasts longer than that of phenol. The use of alcohol was also excluded in the case presented here due to the risks of vasospasm and thrombosis of the vessels adjacent to the superior hypogastric plexus.^[12]^ In addition, conventional radiofrequency leads to a neurodestructive process via an alternating current of electrical energy in the 500 kHz range that yields kinetic energy through Brownian motion (activation and oscillation of the ions in tissue electrolytes).^[13]^ As opposed to the above-mentioned invasive modalities, PRF treatment provides nondestructive neuromodulation without the side effect of denervation and its resultant complications by allowing dispersal of the heat generated by oscillatory motion via vascular runoff during a short rest period (0.5 ms). Hence, as the least invasive procedure, PRF treatment seemed to be the most appropriate for patient safety in the case described here, particularly given the lack of scientific evidence regarding superior hypogastric plexus denervation.

Recently, superior hypogastric neurolysis was used to treat interstitial cystitis and was compared to bladder hydrodistension performed under spinal anesthesia in a prospective randomized setting.^[14]^ In this study, 15 mL of 70% alcohol was used for neurolysis of the superior hypogastric plexus in 12 of 14 patients, among whom 2 patients were excluded due to diagnostic blocks that failed to relieve the pain. During the 2 weeks after the procedure, significant improvements in the symptom score, VAS pain score, and number of daytime frequency and nocturia episodes were observed compared with the baseline values. However, the improvements were not present at the 4-week follow-up because the participants in the study appeared to have abstained from taking analgesic medications following random allocation to the study groups. In contrast, the patient in the case described here was administered a daily dose of gabapentin until the last follow-up. The differences in the mechanisms of action and the durations of the clinical effects of alcohol and PRF treatment might have also contributed to the discrepancies in the clinical course between this case report and the previous study.

In summary, based on our experience, PRF treatment of the superior hypogastric plexus may be used as an alternative treatment option to provide long-term improvements in pain and lower urinary tract symptoms unresponsive to oral medication and hydrodistension of the bladder in interstitial cystitis patients. However, a prospective randomized controlled study is warranted to confirm the clinical efficacy and safety of this procedure for the treatment of interstitial cystitis.
